# Prostate tumor–mediated IFNG signaling primes myeloid cells in bone premetastatic niche for immunosuppressive IL-10 signaling

**DOI:** 10.1172/JCI196347

**Published:** 2025-08-28

**Authors:** Mindy K. Graham, Sarki A. Abdulkadir

**Affiliations:** Department of Urology, Feinberg School of Medicine, Northwestern University, Chicago, Illinois, USA.

**Keywords:** Cell biology, Oncology, Prostate cancer, Transcriptomics

**To the Editor:** Bone is the most common site for metastatic prostate cancer (1). Paradoxically, while the bone marrow is the primary site of immune cell production, prostate cancer cells have evolved effective strategies to evade the immune system and successfully colonize the bone. We sought to understand how prostate cancer cells can prime the bone marrow before metastatic colonization (i.e., the premetastatic niche).

We analyzed a published scRNA-Seq dataset of tissues from patients with prostate cancer and cancer-free individuals (1). This dataset consisted of metastatic tumors of the bone, tumor-adjacent bone marrow, tumor-free bone marrow distant from tumor sites, and benign bone marrow from cancer-free individuals (Supplemental Figure 1A; supplemental material available online with this article; https://doi.org/10.1172/JCI196347DS1). We reasoned that the tumor-free bone marrow from patients with prostate cancer reflects the premetastatic niche bone marrow, harboring alterations conducive to metastatic colonization. We thus compared the benign bone marrow composition with the premetastatic niche bone marrow. Cell proportion analysis showed that erythrocytes, myeloid cells, and NK cells were significantly expanded, while B cells were significantly depleted (Supplemental Figure 1, B and C; Supplemental Table 1; and Figure 1A). Notably, only myeloid cells were enriched in metastatic tumors, tumor-adjacent bone marrow, and premetastatic niche bone marrow (Figure 1B and Supplemental Figure 1, D–F). Expansion of myeloid cells is a recognized feature of the prostate tumor microenvironment (TME) (2). To explore the factors driving myeloid cell enrichment in the premetastatic niche, we performed a hypergeometric analysis of upregulated genes in the myeloid population of the premetastatic niche compared with benign bone marrow. The interferon-γ (IFNG) response pathway emerged as the top Hallmark gene set (Figure 1C). Gene set enrichment analysis further verified that the IFNG response genes were significantly enriched in the myeloid population of the premetastatic niche (Supplemental Figure 2, A and B). This is consistent with previous reports demonstrating that IFNG is sufficient to drive monopoiesis (3).

To identify sources of IFNG responsible for upregulating IFNG signaling in the myeloid cells within the premetastatic niche, we examined ligand and receptor expression across all cell types and samples. We found that *IFNG* was most significantly expressed in T cells (log_2_FC = 1.9, adjusted [adj] *P* = 3.8 × 10^–30^), while *IFNGR1* (log_2_FC = 2.1, adj *P* < 1 × 10^–100^) and *IFNGR2* (log_2_FC = 3.3, adj *P* < 1 × 10^–100^) were most significantly expressed in myeloid cells (Figure 1D and Supplemental Dataset 1). Stratifying by sample revealed that T cells from metastatic tumors, not premetastatic niche or tumor-adjacent bone marrow samples, significantly expressed *IFNG* (log_2_FC = 4.9, adj *P* < 1 × 10^–100^, Figure 1E). Consistent with the IFNG positive feedback loop (4), we observed a significant upregulation of *IFNGR1* in myeloid cells of premetastatic niche bone marrow (Figure 1F).

To assess whether primary tumors can also induce IFNG signaling in the premetastatic niche, we examined scRNA-Seq data of peripheral zone tissues from prostatectomies obtained from men with histologically confirmed prostate cancer (18 samples from 10 men) (5), referred to here as “primary tumor.” Differential gene expression analysis verified that T cells of primary tumors significantly expressed IFNG (log_2_FC = 7.1, adj *P* < 1 × 10^–100^, Supplemental Figure 3A). Correlation analysis of *IFNG* expression in primary tumors indicated that *IFNG*-expressing T cells also expressed chemokine *CCL4* and *CD69*, a marker of T cell activation (Supplemental Figure 3, B–E, and Supplemental Dataset 2). Cell composition analysis, with the healthy prostate included (4), showed that T cells were significantly enriched in primary tumors compared with the healthy prostate (Supplemental Figure 3, F and G, and Supplemental Methods).

IFNG is recognized as a pro-inflammatory cytokine; however, the prostate TME is known to be immunosuppressive (2). To understand how IFNG signaling may contribute to an immunosuppressive TME, we examined the IFNG response genes that were significantly upregulated in myeloid cells of the premetastatic niche (Supplemental Figure 2B and Supplemental Dataset 3). IL-10 receptor-α (*IL10RA*), which encodes a subunit of a receptor activated by the potent antiinflammatory cytokine IL-10, was significantly upregulated in myeloid cells of metastatic tumors, tumor-adjacent bone marrow, and premetastatic niche bone marrow (Figure 1G). This suggests that myeloid cells in the premetastatic niche are primed for IL-10 signaling. Supporting this, our analysis of metastatic tumors revealed that myeloid cells had a significant increase in *IL10* expression (Figure 1H). Additionally, the negative regulator of cytokine signaling *SOCS3*, which acts downstream of IL-10 signaling, was also significantly upregulated in myeloid cells from metastatic tumors (Supplemental Figure 2C). *SOCS3* is a prototypical IL-10 response gene crucial for mediating antiinflammatory activity (6).

In conclusion, our analysis supports a model in which IFNG-expressing T cells within both primary and metastatic prostate tumors systemically upregulate IFNG signaling in bone marrow myeloid cells of the premetastatic niche. This signaling promotes monopoiesis and primes myeloid cells for IL-10–mediated immunosuppressive responses. Consequently, the bone marrow microenvironment is conditioned to support immune evasion before metastatic colonization. While these findings are consistent with a systemic cytokine-driven mechanism, additional in vivo studies are needed to validate this model.

## Funding support

This work is the result of NIH funding, in whole or in part, and is subject to the NIH Public Access Policy. Through acceptance of this federal funding, the NIH has been given a right to make the work publicly available in PubMed Central.

- NIH/National Cancer Institute grants R01CA257258 (awarded to SAA) and P50CA180995 (PI: SAA. Career enhancement program awarded to MKG).

## Supplementary Material

Supplemental data

Supplemental data sets 1-3

Supporting data values

## Figures and Tables

**Figure 1:**
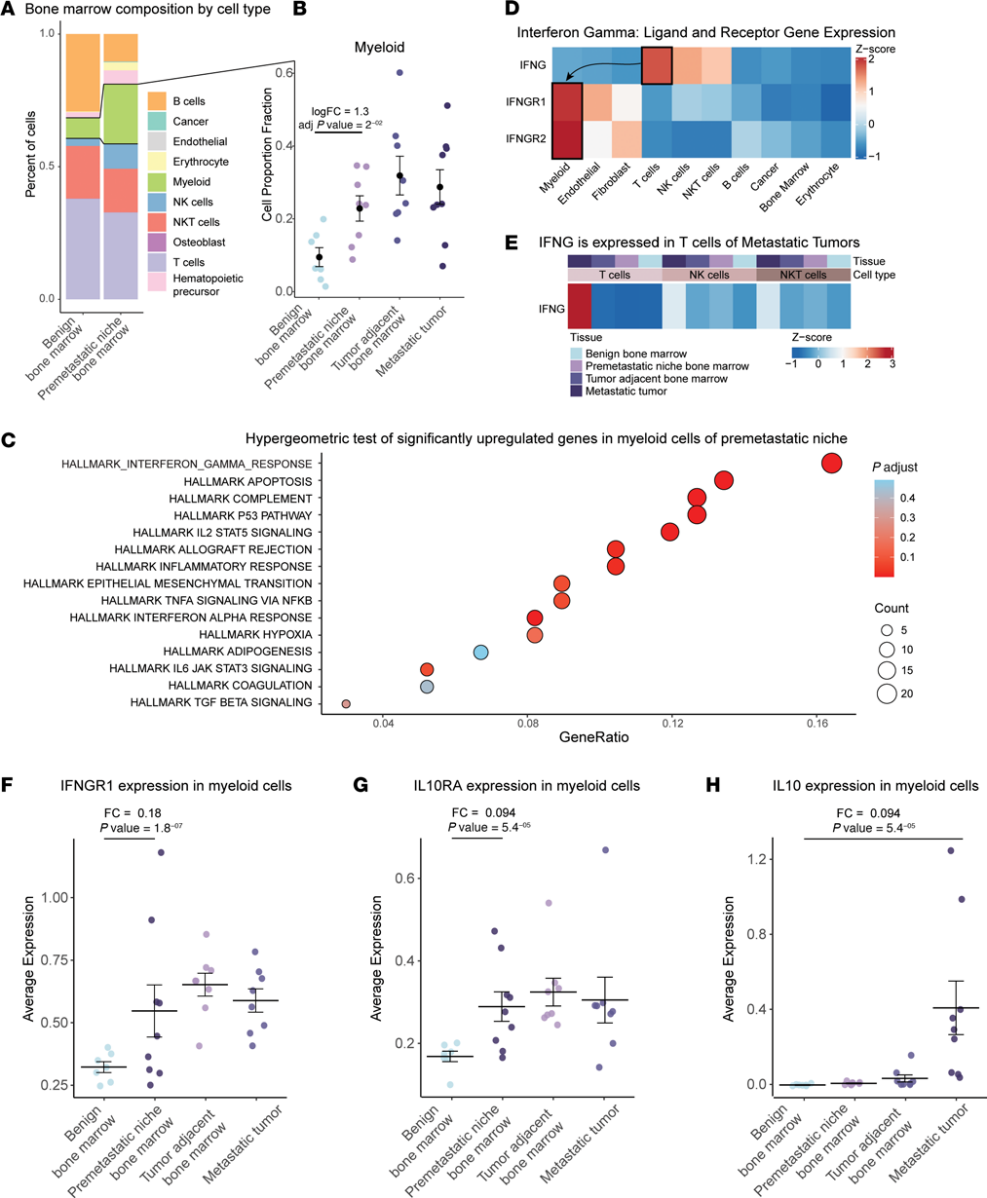
IFNG signaling is upregulated in bone marrow myeloid cells of the premetastatic niche in patients with prostate cancer. (**A**) Stacked bar plot showing the percentage composition of cell types in benign (12,400 cells) and premetastatic niche bone marrow (18,259 cells). (**B**) Scatterplot of myeloid cell fraction of benign bone marrow (*N* = 7), premetastatic niche bone marrow (*N* = 8), tumor-adjacent bone marrow (*N* = 8), and metastatic tumor (*N* = 9). (**C**) Hypergeometric test of significantly (*P* < 0.05) upregulated genes in myeloid cells of the premetastatic niche compared with benign bone marrow. Heatmaps showing (**D**) *IFNG* and receptor expression (*IFNGR1*, *IFNGR2*) across all cells and (**E**) *IFNG* expression of lymphoid cells grouped by tissue type. Scatterplots of average expression of (**F**) *IFNGR1*, (**G**) *IL10RA*, and (**H**) *IL10* in myeloid cells across bone marrow and metastatic tumor samples. Bars represent mean ± SEM. Cell proportions and differential gene expression analysis were conducted using RAISIN (version 1) to generate log fold-change and *P* values to determine significance.

## Version 1 08/28/2025

In-Press Preview

## Version 2 11/03/2025

Electronic publication
