# Supplementary material for: Prostate tumor–mediated IFNG signaling primes myeloid cells in bone premetastatic niche for immunosuppressive IL-10 signaling
Source: J Clin Invest. 2025 Aug 28;135(21):e196347. doi: 10.1172/JCI196347 (PMC12578376; doi:10.1172/JCI196347)
Supplement: Supplemental data [file jci-135-196347-s051.pdf]

SUPPLEMENTAL MATERIAL

FIGURES

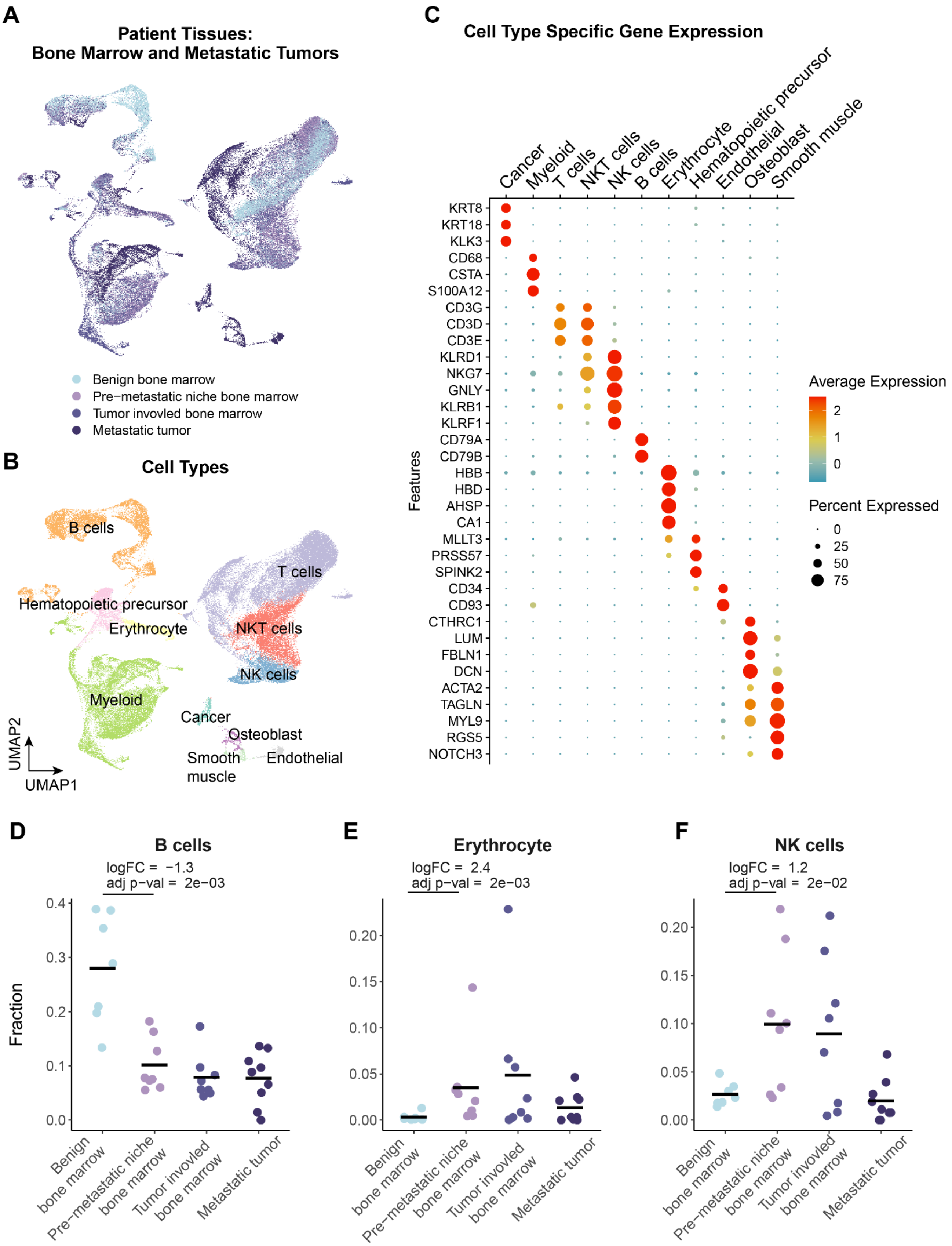

Supplemental Figure S1. Reanalyzing the scRNA-seq dataset of tissue samples collected from patients diagnosed with metastatic prostate cancer and cancer-free individuals (Kfoury et al. 2021). Dimensionality reduction UMAPs of **(A)** samples collected from bone metastasis (metastatic tumor), tumor-adjacent bone marrow, bone marrow distant from tumor sites (premetastatic niche bone marrow), and bone marrow from cancer-free individuals (benign bone marrow), and **(B)** annotated cell types. **(C)** A dot plot indicating cell-type-specific gene expression across clusters. Scatterplots of **(D)** B cell, **(E)** erythrocyte, and **(F)** NK cell fractions across bone marrow and bone metastasis samples.

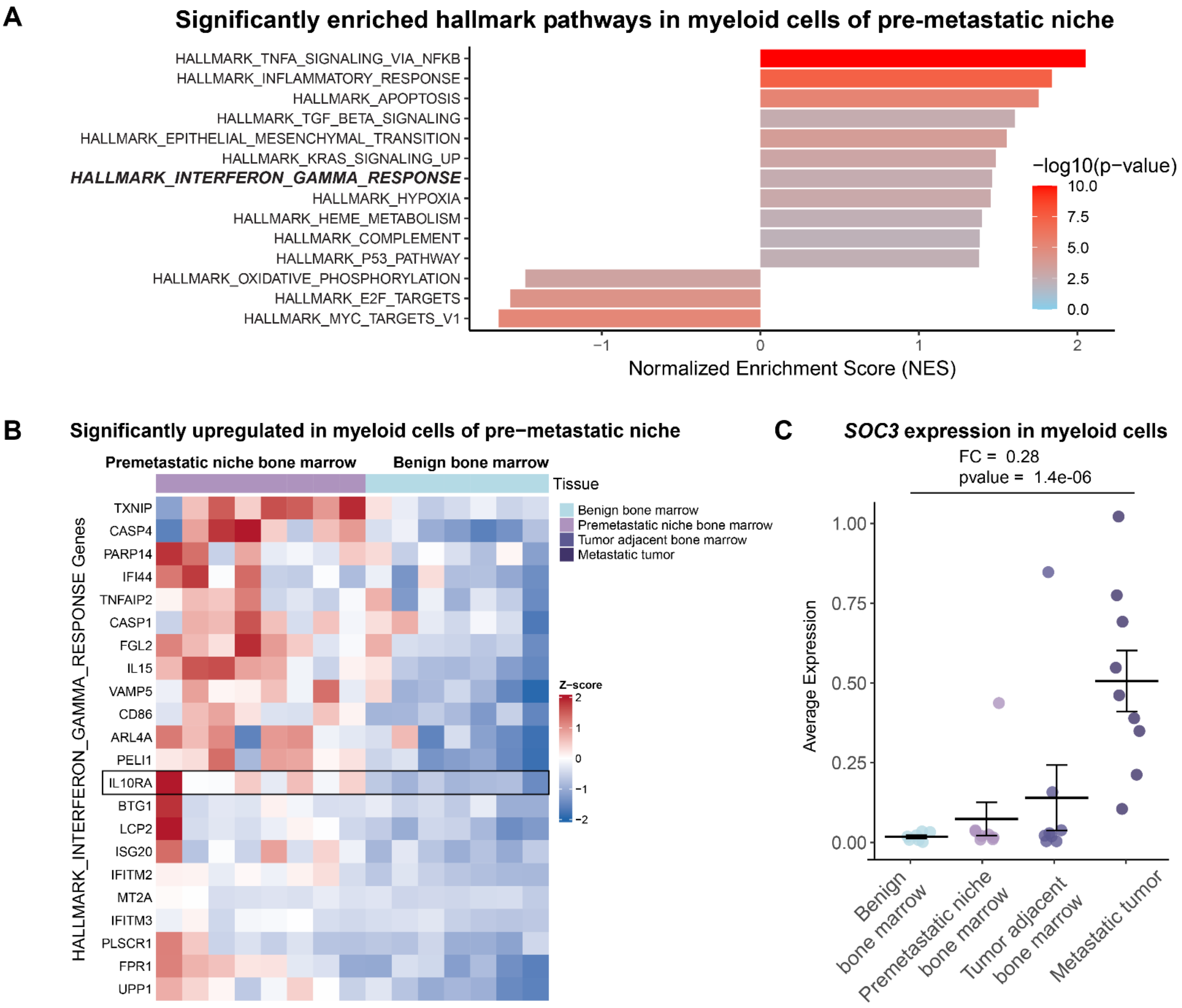

Supplemental Figure S2. Myeloid cells in the premetastatic niche are primed for immunosuppressive IL-10 signaling. (A) Bar graph showing all significant gene sets of the hallmark collection identified through gene set enrichment analysis (GSEA) when comparing myeloid cells from the premetastatic niche to those from benign bone marrow. **(B)** Heatmap of significantly upregulated IFNG response genes in myeloid cells of the premetastatic niche versus benign bone marrow with *IL10RA* highlighted. **(C)** Scatterplot showing the average *SOCS3* expression in myeloid cells across bone marrow and bone metastasis samples. *SOCS3* is significantly upregulated in myeloid cells from metastatic tumors.

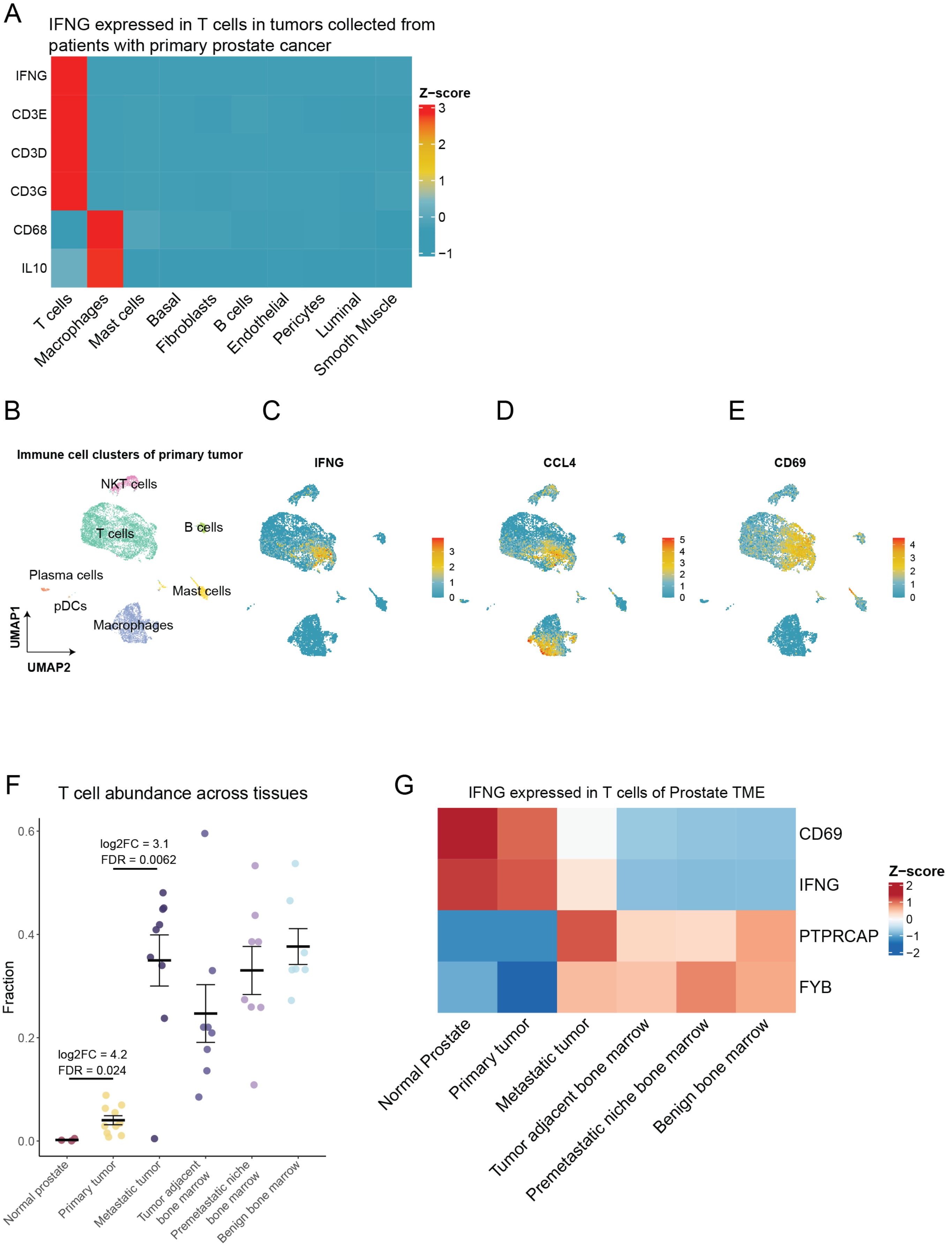
A
IFNG expressed in T cells in tumors collected from patients with primary prostate cancer
IFNG
CD3E
CD3D
CD3G
CD68
IL10
Z−score
3
2
1
0
−1
T cells
Macrophages
Mast cells
Basal
Fibroblasts
B cells
Endothelial
Pericytes
Luminal
Smooth Muscle
B
Immune cell clusters of primary tumor
NKT cells
T cells
B cells
Plasma cells
Mast cells
pDCs
Macrophages
UMAP1
UMAP2
C
IFNG
D
CCL4
E
CD69
F
T cell abundance across tissues
Fraction
log2FC = 3.1
FDR = 0.0062
log2FC = 4.2
FDR = 0.024
Normal prostate
Primary tumor
Metastatic tumor
Tumor adjacent bone marrow
Premetastatic niche bone marrow
Benign bone marrow
G
IFNG expressed in T cells of Prostate TME
CD69
IFNG
PTPRCAP
FYB
Z−score
Normal Prostate
Primary tumor
Metastatic tumor
Tumor adjacent bone marrow
Premetastatic niche bone marrow
Benign bone marrow

Supplemental Figure S3. IFNG is expressed in a subset of T cells in human primary prostate cancer. **(A)** Heatmap of prostatectomy scRNA-seq data grouped by cell type showing *IFNG* expressed in T cells, and *IL10* expressed in macrophages. Dimensionality reduction UMAPs of **(B)** the immune populations subsetted from prostatectomy scRNA-seq data, showing a subset of T cells express **(C)** *IFNG*, **(D)** *CCL4*, and **(E)** *CD69*. **(F)** Scatterplot of T cell fraction of normal prostate (N = 3), benign bone marrow (N = 7), premetastatic niche bone marrow (N = 8), tumor-adjacent bone marrow (N = 8), and metastatic tumor (N = 9). Cell proportions were analyzed using RAISIN (version 1) to generate log fold change and p-values to determine significance. (G) Heatmap showing *IFNG* expression of T cells (as defined by CD3 expression) grouped by tissue type. *PTPRCAP* and *FYB* are expressed in T cell-committed lymphocytes of the bone marrow.

SUPPLEMENTAL TABLES

Table S1. Results of the cell proportion test comparing the bone marrow compositions of the premetastatic niche and benign samples.

Cluster	logFC	P.Value	adj.P.Val
Erythrocyte	2.43	2.96E-04	2.25E-03
Myeloid	1.26	6.71E-03	1.68E-02
NK cells	1.18	6.67E-03	1.68E-02
Hematopoietic precursor	0.92	8.54E-02	1.71E-01
Cancer	0.71	2.01E-01	2.87E-01
Endothelial	-0.09	6.76E-01	6.76E-01
T cells	-0.26	4.09E-01	4.54E-01
Osteoblast	-0.39	3.04E-01	3.80E-01
NKT cells	-0.64	1.12E-01	1.86E-01
B cells	-1.27	4.50E-04	2.25E-03

SUPPLEMENTAL INFORMATION

We compared the T cell abundance (CD3+ cells) in normal prostate (1), primary and metastatic tumors, and bone marrow (Supplemental Figure S3F). T cells are rare in the normal prostate (0.21% +/- 0.13%). Compared to normal prostate, there is a significant increase in T cells in primary tumors (log2FC = 4.2, FDR = 0.024). The T cells of metastatic tumors of the bone are also significantly enriched compared to primary tumors (log2FC = 3.2, FDR = 0.0062). However, the CD3+ cells of metastatic tumors likely reflect a mixture of fully differentiated activated T cells (CD69+) and T cell-committed lymphocytes of the bone marrow (PTPRCAP+/FYB+, Supplemental Figure S3G). T cells of primary tumors express more *IFNG* than metastatic tumors (log2FC = 0.9, adjusted p-value = 1.1e-27), further supporting the potential of primary tumors to upregulate IFNG signaling in the myeloid cells of the premetastatic niche bone marrow.

METHODS

Sex as a biological variable

As prostate cancer is a disease specific to men, sex was not considered a biological variable for this analysis.

Analysis of scRNA-seq data

Expression datasets generated from prostate cancer bone metastasis and bone marrow samples, as described by Kfoury et al., were accessed through NCBI GEO (GSE143791) (2). ScRNA-seq gene by cell count matrices for each sample were aggregated and normalized using variance stabilization with Seurat (version 5.0.3) SCTransform (version 2), which employs a gamma-Poisson generalized linear model fitting (3, 4). For dimensionality reduction and clustering analysis, a principal component analysis was performed, and 40 principal components were used to compute the Uniform Manifold Approximation and Projection (UMAP) dimensions and perform Louvain clustering at a resolution of 0.3. Differential gene expression analysis of previously characterized cell-type-specific genes was used to identify the cell type for each cluster (5). The expression datasets of prostatectomy samples that we previously described (5) (NCBI dbGaP accession code phs003480.v1.p1) were reanalyzed to examine *IFNG* and *IL10* gene expression in the tumor microenvironment (TME) of primary prostate cancer. Normal prostate scRNA-seq data from Henry et al. were reanalyzed for cell proportion and gene expression analysis of T cells (GEO: GSE120716) (1).

Cell proportion test

To assess if cell type composition changed across sample types, we applied linear regression analysis (limma) in scRNA-seq with multiple samples (RAISIN, version 1.0) (6). A cell proportions test was performed between benign bone marrow and one of the tissues from patients with prostate cancer (bone metastases, bone marrow from tumor-involved sites, and bone marrow distant from tumor sites). The adjusted p-value was derived from a two-sided t-test and adjusted for multiple hypothesis testing using the Benjamini-Hochberg procedure.

Differential gene expression and pathway analysis

Differential expression between cell types and or tissue types was performed using either Seurat's FindMarkers function (3) or RAISIN's raisintest function (6). Genes were ranked by their signed statistic. Gene sets from the Molecular Signatures Database (MSigDB) were imported using the R package msigdbr (version 7.5.1) (7). Gene over-presentation was assessed using clusterProfiler (version 4.14.6) enricher function. Specifically, genes with a fold change > 0 and a p-value of < 0.05 were selected for the hypergeometric test (8). In contrast, no statistical cutoff was used to perform gene set enrichment analysis (GSEA). All genes and their associated statistic were the input for fgsea (version 1.22.0) to calculate normalized enrichment scores (NES) and adjusted p-values (9). Outputs from differential gene expression and pathway analysis were utilized to create heatmaps, bar plots, and scatter plots. To determine genes that co-express with *IFNG*, the T cell was subclustered from the prostatectomy scRNA-seq dataset (phs003480.v1.p1). Pearson correlation analysis of *IFNG* across all genes in T cells was performed (Supplemental Data S2).

Data and code availability

The human prostatectomy data we previously generated and used in this analysis can be accessed through NCBI dbGaP under accession code phs003480.v1.p1 (5). Access to data can be requested using the following link: https://www.ncbi.nlm.nih.gov/projects/gap/cgi-bin/study.cgi?study_id=phs003480.v1.p1. The scRNA-seq expression datasets generated from prostate cancer bone metastasis and bone marrow samples, as described by Kfoury et al., can be accessed through NCBI GEO (GSE143791) (2). Normal prostate scRNA-seq data from Henry et al. can be accessed through NCBI GEO (GSE120716) (1). All code used to perform the analysis and generate the outputs can be found as Rmd files or knitted HTML files on a GitHub repository https://github.com/MKGraham-Group/IFNG_premetastatic_PCa.git. The supporting data values file contains the cell type composition data (supporting data 1) and myeloid expression of *IFNGR1*, *IL10RA*, *IL10*, and *SOCS3* (supporting data 2).

Statistics

Cell proportions and differential gene expression analysis were conducted using RAISIN (version 1) (5). RAISIN uses a hierarchical linear mixed-effects regression model, specifically designed for single-cell RNA-seq

datasets with multiple samples. P-values for cell proportions were derived from an unpaired mixed-effects regression framework. For differential gene expression, p-values were obtained using a hierarchical linear mixed model with empirical Bayes shrinkage for variance estimation and inference. The adjusted p-values were calculated to correct for multiple hypothesis testing using the Benjamini-Hochberg procedure for both cell proportions and differential gene expression analyses.

Study approval

All human data analyzed in this study were obtained from publicly available datasets from previously published studies. The prostatectomy data generated by our group are available through NCBI dbGaP (accession: phs003480.v1.p1). The bone metastasis and bone marrow scRNA-seq data from Kfoury et al. are available through NCBI GEO (GSE143791). The normal prostate scRNA-seq data from Henry et al. are available through NCBI GEO (GSE120716). As these datasets were previously collected and de-identified before public release, additional institutional review board (IRB) approval and informed consent were not required for this secondary analysis.

AKNOWLEDGEMENTS

This work was supported in part by NIH/NCI grants R01CA257258 (awarded to S.A.) and P50CA180995 (PI: S.A. career enhancement program awarded to M.K.G.).

SUPPLEMENTAL REFERENCES

1. Henry GH, et al. A Cellular Anatomy of the Normal Adult Human Prostate and Prostatic Urethra. *Cell Rep*. 2018;25(12):3530–3542.e5.

2. Kfoury Y, et al. Human prostate cancer bone metastases have an actionable immunosuppressive microenvironment. *Cancer Cell*. 2021;39(11):1464–1478.e8.

3. Hao Y, et al. Dictionary learning for integrative, multimodal and scalable single-cell analysis. *Nat Biotechnol*. 2024;42(2):293–304.

4. Choudhary S, Satija R. Comparison and evaluation of statistical error models for scRNA-seq. *Genome Biol*. 2022;23(1):27.

5. Graham MK, et al. Convergent alterations in the tumor microenvironment of MYC-driven human and murine prostate cancer. *Nat Commun*. 2024;15(1):7414.

6. Ji Z. Statistical Methods for Decoding Gene Regulation in Single Cells. 2020.

7. Liberzon A, et al. The Molecular Signatures Database (MSigDB) hallmark gene set collection. *Cell Syst*. 2015;1(6):417–425.

8. Yu G, et al. clusterProfiler: an R package for comparing biological themes among gene clusters. *OMICS*. 2012;16(5):284–287.

9. Sergushichev. An algorithm for fast preranked gene set enrichment analysis using cumulative statistic calculation. *bioRxivorg*. https://www.researchgate.net/profile/Alexey-Sergushichev/publication/304251126_An_algorithm_for_fast_preranked_gene_set_enrichment_analysis_using_cumulative_statistic_calculation/links/576a910308aefcf135bd206c/An-algorithm-for-fast-preranked-gene-set-enrichment-analysis-using-cumulative-statistic-calculation.pdf.
